# Locally Recurrent Rectal Cancer in the Lateral Compartment: Imaging Features and Association with Primary Tumour Characteristics

**DOI:** 10.1245/s10434-025-19068-w

**Published:** 2026-01-22

**Authors:** F. E. C. Vande Kerckhove, D. M. J. Creemers, E. Banken, S. H. J. Ketelaers, R. R. J. Coebergh van den Braak, G. A. P. Nieuwenhuijzen, A. E. Verrijssen, A. W. Daniëls-Gooszen, T. R. van Oudheusden, S. G. van Ravensteijn, I. E. G. van Hellemond, H. J. T. Rutten, H. M. U. Peulen, J. G. Bloemen, J. Nederend, J. W. A. Burger

**Affiliations:** 1 Department of Surgery, Catharina Hospital Eindhoven, Eindhoven, The Netherlands; 2 GROW Research Institute for Oncology and Reproduction, Maastricht University, Maastricht, The Netherlands; 3 Department of Medical Oncology, Catharina Hospital Eindhoven, Eindhoven, The Netherlands; 4 Department of Radiation Oncology, Catharina Hospital Eindhoven, Eindhoven, The Netherlands; 5 Department of Radiology, Catharina Hospital Eindhoven, Eindhoven, The Netherlands

**Keywords:** Locally recurrent rectal cancer, Imaging, Lateral lymph nodes, Surgery, Survival

## Abstract

**Background and Purpose:**

Locally recurrent rectal cancer (LRRC) involving the lateral pelvic compartment is associated with a poor prognosis. The underlying aetiology, such as lateral lymph node(s) (LLN) metastases or other high-risk features, remains unclear. This study aimed to investigate features of lateral LRRC and their anatomical association with primary tumour characteristics.

**Methods:**

All patients with lateral LRRC referred to our centre between 2018 and 2025 were included (*n *= 104). Primary and recurrent tumour MRIs were centrally re-reviewed to evaluate tumour features. An expert panel further assessed anatomical relationships between the primary tumour locations and lateral LRRC sites. Primary outcome was to characterise recurrence features; secondary outcomes were overall and local re-recurrence-free survival.

**Results:**

Primary tumours of 104 patients frequently showed a high prevalence of high-risk radiological features: extramural vascular invasion (45%), cT3cd–cT4ab staging (47%), tumour deposits (23%) and pathological LLNs (≥ 7 mm short axis, LLN+, 20%). In patients with LLN+ primary tumours (*n *= 21), the incidence of the other synchronous high-risk features was significantly increased. Within this group, primary LLN dissection (LLND) was performed in 7/21 (33%). Subsequent lateral LRRC due to LLN nodal recurrence occurred in 10/21 (48%), including 4 patients who had undergone LLND. Overall, the expert panel linked 11% of lateral LRRC to LLNs visible on primary imaging, and the remaining 89% to other causes.

**Conclusions:**

Only a minority of lateral LRRCs could be attributed to primary LLN+. Other causes, related to primary tumour spread and high-risk features, appear to be associated with the majority of lateral LRRC, suggesting a multifactorial aetiology.

**Supplementary Information:**

The online version contains supplementary material available at 10.1245/s10434-025-19068-w.

Locally recurrent rectal cancer (LRRC) involving the lateral pelvic compartment(s), defined as the lateral wall musculature, soft tissue of the pelvic sidewall, pelvic lymph nodes, iliac vessels, sacral nerve plexus or lateral bony pelvis, accounts for approximately 18–50% of local recurrences.^1–6^ Lateral LRRC is associated with poorer overall survival (OS) and disease-free survival (DFS) compared with central recurrences.^1,4,7–9^ The underlying mechanisms contributing to lateral recurrences, however, remain insufficiently understood.

One of the implicated potential risk factors of lateral LRRC is the presence of lateral (pelvic) lymph nodes (LLN) metastases in distal primary rectal cancer, defined as the involvement of internal iliac and obturator lymph nodes.^10–12^ For primary rectal cancer patients with primarily enlarged LLN (short axis (SA) ≥ 7 mm) undergoing neoadjuvant (chemo)radiotherapy with total mesorectal excision (TME) surgery, a 5-year lateral LRRC rate ranging between 17.9% to 19.5% has been observed,^13–15^ compared with the generally reported rates of 5–10%. These observations have contributed to growing interest in prophylactic or selective LLN dissection (LLND), though its role remains controversial with substantial clinical practice variation between Western and Eastern centres.^16^

In particular, it remains uncertain whether the majority of lateral recurrences primarily result from LLN involvement. Other tumour-related features, including (y)pT stage, tumour deposits, extramural vascular invasion (EMVI) and mesorectal fascia (MRF) invasion, have also been associated with increased LRRC rates.^17,18^ Consequently, a clearer understanding of the causes of lateral LRRC is needed to optimise treatment strategies.

This study aimed to characterise the radiological features of lateral LRRC, including assessment of their anatomical relationship to primary tumour features in a large retrospective cohort. Additionally, we report OS and local re-recurrence-free survival (LRFS) in patients with lateral LRRC.

## Patients and Methods

### Patient Selection

All patients with LRRC treated at Catharina Hospital Eindhoven, a national tertiary referral centre treating a substantial proportion of LRRC cases in the Netherlands, are prospectively registered in a database. All patients diagnosed with lateral LRRC, between January 2018 and January 2025, were retrospectively identified and reviewed from this database. Patients for whom baseline or restaging MRI was not available for the primary and/or recurrent tumour were excluded. Baseline and radiological characteristics, treatment intention, neoadjuvant therapy, surgery and oncological outcomes were recorded. The cut-off date for follow-up was May 1st, 2025. This study was approved by the Dutch Medical Research Ethics Committees United – Nieuwegein (registration no. AW24.062/W23.008).

### Neoadjuvant Therapy and Surgery

For the primary tumour, patients received neoadjuvant therapy according to the standard Dutch clinical practice guidelines applicable between 2012 and 2022. TME surgery alone was recommended for early rectal tumours (cT1–3ab(MRF-)N0). For intermediate-risk rectal tumours (cT3cdN0 or cT1–3(MRF-)N1), neoadjuvant short course radiotherapy (SCRT, 5 × 5 Gy) followed by TME surgery was discussed. For locally advanced rectal cancer (LARC) (cT4 and/or cN2 and/or EMVI and/or MRF+ and/or extra-mesorectal lymph node(s)), treatment focused on neoadjuvant chemoradiotherapy (CRT, 25 × 5 Gy with concomitant capecitabine) followed by TME surgery. A LLND was performed (between 2012 and 2021, outside of formal study setting), generally for enlarged LLNs (SA ≥ 7 mm) on primary MRI. Selected LARC patients received induction chemotherapy (ICT) prior to (chemo)radiotherapy, which generally consisted of three or four courses of capecitabine and oxaliplatin (CAPOX) or four to six courses of 5-FU, leucovorin and oxaliplatin (FOLFOX) or 5-FU, leucovorin, oxaliplatin and irinotecan (FOLFOXIRI).

Patients with resectable lateral LRRC without metastatic disease eligible for treatment with curative intent were generally scheduled for neoadjuvant chemo(re)irradiation and surgery, as determined by our multidisciplinary team (MDT). Radiotherapy-naïve patients were planned for full-course CRT, consisting of a cumulative dose of 50 Gy in 25 fractions of 2 Gy targeting tumour and elective volumes. In previously irradiated patients, chemo-reirradiation was planned, consisting of a cumulative dose of 30 Gy in 15 fractions of 2 Gy targeting the tumour only. Selected patients with LRRC received ICT prior to chemo-(re)irradiation, which generally consisted of three or four courses of capecitabine and oxaliplatin (CAPOX) or four to six courses of 5-FU, leucovorin and oxaliplatin (FOLFOX) or 5-FU, leucovorin and irinotecan (FOLFIRI). Type and extent of surgery was determined during the MDT. In case of concerns for microscopic residual disease, intraoperative electron beam radiotherapy (IOERT) was delivered in a single dose of 10–12.5 Gy. Since 2021, a dose-adapted technique has allowed delivery of higher surface doses between 15.5 and 16.5 Gy. Patients with unresectable lateral LRRC, extensive metastatic disease or poor clinical condition underwent palliative treatment. Owing to lack of palliative LRRC guidelines, treatment varied on the basis of patient presentation, previous treatment regimen, clinical condition, symptomatic burden or extent of disease. Therapy consisted of palliative radiotherapy for local disease and symptomatic relief, systemic therapy, chemo(radiotherapy) and best supportive care.

### Central Radiological Assessment

**Fig 1:**
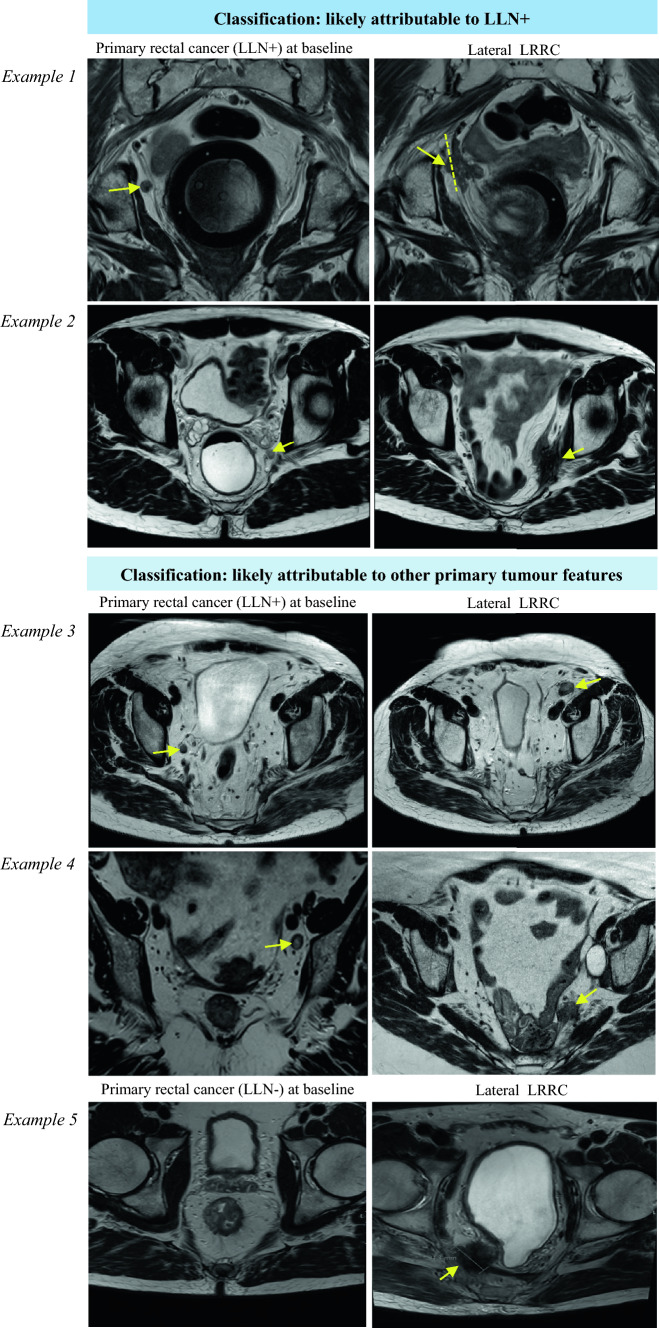
Illustrative examples of side-to-side radiological review to determine anatomical relationship between lateral LRRC and primary tumour, as determined in expert panel review. *LRRC* locally recurrent rectal cancer, *LLN+* pathological lateral lymph node

MRI was performed for both primary and recurrent tumours at baseline and, when applicable, after neoadjuvant therapy. MRI imaging acquisition adhered to the standard Dutch clinical practice guidelines applicable at the time of diagnosis, and consisted of at least T2-weighted high-resolution sequences (1.5 or 3.0 Tesla) in true sagittal, coronal and transverse plans and diffusion-weighted imaging (DWI) in axial plane. All MRIs were retrieved from institutional or referral archives. All MRIs were re-reviewed independently by experienced abdomen radiologists from our tertiary care centre with specific expertise in LRRC according to a systematic report. MRI reassessment of primary and recurrent tumours covered staging, treatment response and anatomical and compartmental involvement based on the European Society of Gastrointestinal and Abdominal Radiology (ESGAR) rectal cancer template for primary tumours and a standardised reporting checklist for recurrent disease (Supplementary Information 1). An expert panel, comprising two experienced surgical oncologists (J.W.B., J.B.) and two radiologists (J.N., T.H.) with LRRC expertise, further radiologically assessed the anatomical relationship between the primary tumour location and the subsequent site of lateral LRRC. For each case, the likely aetiology of recurrence, whether likely attributable to LLNs or other causes relating to the primary tumour (i.e. tumour spread or high-risk features), was determined through unanimous consensus among all four reviewers. This involved side-by-side review of the primary and recurrent MRIs. Recurrence was classified as likely related to LLN nodal disease if there was a clear spatial concordance between the recurrence site and anatomical location of previously identified LLN(s), based on radiological overlay. If the recurrence was located in a distinct anatomical location unrelated to the primary LLN(s), it was classified as likely attributable to other primary tumour causes. In the event of disagreement or uncertainty, all four panel members re-reviewed the MRI together to reach consensus. Representative cases illustrating this classification methodology are demonstrated in Fig. 1.

### Outcomes

The primary study outcome was to characterise the radiological features of lateral LRRC, including assessment of their anatomical relationship to primary tumour characteristics to explore potential causal pathways. Secondary outcomes were OS and LRFS of patients with lateral LRRC. For all patients, OS was calculated from date of LRRC diagnosis until date of death or censored at last follow-up. For patients with LRRC who were surgically treated, OS was calculated from date of surgery until date of death or censored at last follow-up, and LRFS was calculated from date of surgery until the date on which local re-recurrence was detected by imaging or histology, or censored at last follow-up, or death.

### Statistical Analysis

Continuous data were calculated and are expressed as median with interquartile range (IQR) or mean (± standard deviation), as appropriate. Categorical data are reported as count and percentages. Group comparisons for categorical data were performed using chi-squared test of Fisher’s exact test, where appropriate. Groups comparisons for continuous data were performed with the Mann–Whitney *U* test. The Kaplan–Meier survival method was used to analyse OS and LRFS. Differences were assessed by employing the long-rank test. A two-sided *p*-value ≤ 0.05 was considered statistically significant. All statistical analyses were performed using SPSS Statistics 29.0 software (IBM, Endicott, NY, USA).

## Results

### Patient Characteristics

**Fig 2:**
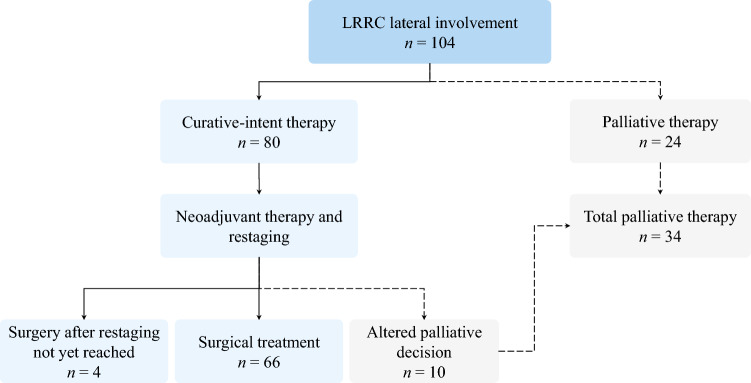
Flowchart of included patients with lateral LRRC undergoing curative-intent and palliative therapy at baseline in our institution. LRRC, locally recurrent rectal cancer

A total of 104 patients were included. At diagnosis of lateral LRRC, 80 (77%) patients were considered eligible for curative-intent therapy, and 24 (23%) received palliative treatment (Fig. 2) owing to distant metastasis (71%), extensive tumour involvement in ≥ 3 compartments (75%) and/or multifocal disease (67%). Among those treated with curative intent, 66 underwent surgery, 4 were awaiting surgery at last follow-up, and 10 patients were treated with palliative treatment after disease progression was identified at response assessment.

### Primary Tumour: Characteristics and Treatment

Primary tumour characteristics of 104 patients with lateral LRRC are presented in Table 1*.* On baseline MRI, primary tumours showed EMVI in 45%, cT3cd–cT4ab staging in 47%, tumour deposits in 23% and pathological LLN (LLN+, ≥ 7 mm) in 20%. The majority of patients received neoadjuvant radiotherapy (58% CRT, 12% SCRT). On restaging MRI, observed EMVI+ decreased to 30% and cT3cd–cT4ab staging to 37%. A partial response of tumour deposits and/or LLN+ was observed in 35%.

**Table 1:** Primary tumour disease characteristics, treatment and histopathological parameters

		Total (*n *= 104) *N* (%)
Gender	Male	71 (68)
	Female	33 (32)
Age at diagnosis primary	Mean, years (SD)	61 (9)
*Baseline MRI, primary*		
Maximum diameter tumour	Median, mm (IQR)	45 (39–59)
Type of tumour	Solid	97 (93)
	Mucinous	7 (7)
EMVI presence	Negative	57 (55)
	Positive (grade 3–4)	47 (45)
Mesorectal fascia	Not involved	57 (55)
	Involved (≤ 1 mm)	19 (18)
	Invasion	28 (27)
Distance anal verge and inferior border tumour	Median, mm (IQR)	43 (13–65)
cT category	cT1-2	13 (13)
	cT3ab	42 (40)
	cT3cd	25 (24)
	cT4ab	24 (23)
cN category	cN0	40 (38)
	cN1abc	32 (31)
	cN2ab	32 (31)
LLN presence, ≥ 7 mm	None	83 (80)
	Single	17 (16)
	Multiple single side	2 (2)
	Both sides	2 (2)
Short-axis LLN	Median, mm (IQR)	10 (8–12)
Tumour deposit(s) presence	Yes	24 (23)
*Neoadjuvant therapy, primary*		
Induction chemotherapy	Yes	16 (15)
Radiotherapy	None	31 (30)
	SCRT (5×5 Gy)	13 (12)
	CRT (25×2 Gy)	60 (58)
Restaging MRI after neoadjuvant therapy (n = 69)*		
Maximum diameter tumour	Median, mm (IQR)	32 (23–44)
EMVI presence	Negative	48 (70)
	Positive (grade 3–4)	21 (30)
Mesorectal fascia	Not involved	41 (59)
	Involved (≤ 1 mm)	12 (17)
	Invasion	16 (23)
ycT category	ycT0–2	18 (26)
	ycT3ab	25 (36)
	ycT3cd	10 (14)
	ycT4ab	16 (23)
ycN category	ycN0	39 (57)
	ycN1abc	27 (39)
	ycN2ab	3 (4)
Short-axis LLN	Median, mm (IQR)	5 (0-8)
Response LLN/tumour deposits	Not applicable	36 (52)
	Complete response	9 (13)
	Partial response	24 (35)
Overall response	Good	36 (52)
	Partial	30 (44)
	Poor	3 (4)
*Treatment, primary*		
Surgical procedure	LAR	60 (58)
	APR	41 (40)
	Exenterative surgery	3 (3)
Intraoperative electron beam radiotherapy	Yes	9 (9)
LLN dissection (*n* = 21)	Yes	7 (33)
Adjuvant therapy	None	99 (95)
	Chemotherapy	5 (5)
pT category	(y)pT0-2	30 (29)
	(y)pT3	66 (63)
	(y)pT4	8 (8)
pN category	(y)pN0	54 (52)
	(y)pN1-2	50 (48)
R status	R0	90 (87)
	R1	12 (12)
	Unknown	2 (2)

IQR, interquartile range; EMVI, extramural vascular invasion; cT, clinical T stage; cN, clinical N stage; LLN, lateral lymph nodes; SCRT, short-course radiotherapy; mrTRG, MRI tumour regression grade; DWI, diffusion-weighted imaging; ycT, post-neoadjuvant treatment T stage; ycN, post-neoadjuvant N stage; LAR, low anterior resection; APR, abdominoperineal resection; pT, pathological T stage; pN, pathological N stage; R0, clear resection margin; R1, irradical resection margin.

*Completion of restaging MRIs after 5×5 Gy varied per referral centre (*n* = 4 patients undergoing RT, restaging was not performed)

**Owing to rounding, not all percentages added up to 100%

### Primary Tumour: Lateral Lymph Node Involvement

Primary tumour characteristics stratified according to LLN presence are summarised in Table 2. In LLN+ primary tumours (*n *= 21), the incidence of other synchronous high-risk features was significantly increased compared with LLN− primary tumours (*n *= 83). LLN+ primary tumours presented with a larger tumour diameter (53 (IQR 45–78) mm versus 43 (IQR 37–55) mm, *p *= 0.004), and more often with EMVI+ (79% versus 39%, *p *= 0.007), MRF involvement/invasion (76% versus 37%, *p *= 0.003), tumour deposits (48% versus 17%, *p *= 0.003) and cT3cd–cT4ab staging (76% versus 40%, *p *= 0.020).

**Table 2:** Primary tumour and lateral LRRC disease characteristics, stratified according to LLN presence

		LLN + primary (*n =* 21)	LLN – primary (*n =* 83)	*p-*Value
Interval primary–LRRC diagnosis	Median, months (IQR)	24 (16–59)	28 (18–47)	0.852
*Primary tumour, baseline MRI*				
Maximum diameter tumour	Median, mm (IQR)	53 (45-78)	43 (37–55)	**0.004**
EMVI presence	Negative	6 (29)	51 (61)	**0.007**
	Positive (3–4)	15 (79)	32 (39)	
Mesorectal fascia	Not involved	5 (24)	52 (63)	**0.003**
	Involved (≤ 1 mm)	8 (38)	11 (13)	
	Invasion	8 (38)	20 (24)	
Distance anal verge and inferior border tumour	Median, mm (IQR)	25 (0–54)	50 (20–69)	**0.020**
cT category	cT1-2	–	13 (16)	**0.020**
	cT3ab	5 (24)	37 (45)	
	cT3cd	8 (38)	17 (21)	
	cT4ab	8 (38)	16 (19)	
LLN presence, ≥ 7 mm	None	–	83 (100)	N/A
	Single	17 (81)		
	Multiple single side	2 (10)		
	Both sides	2 (10)		
Short-axis LLN, baseline	Median, mm (IQR)	10 (8–12)	–	N/A
Tumour deposit(s) present	Yes	10 (48)	14 (17)	**0.003**
*Neoadjuvant therapy, primary*				
Induction chemotherapy	Yes	3 (14)	13 (16)	0.876
Radiotherapy	None	1 (5)	30 (36)	**< 0.001**
	SCRT (5×5 Gy)	–	13 (16)	
	CRT (25×2 Gy)	20 (95)	40 (48)	
*Restaging MRI, primary*		*n *= 20	*n *= 49*	
Maximum diameter tumour	Median, mm (IQR)	38 (26–53)	31 (22–43)	0.108
EMVI presence	Negative	12 (60)	36 (73)	0.270
	Positive (3–4)	8 (40)	13 (27)	
Mesorectal fascia	Not involved	10 (50)	31 (63)	0.618
	Involved (≤ 1 mm)	4 (20)	8 (16)	
	Invasion	6 (30)	10 (20)	
Short-axis LLN	Median, mm (IQR)	4 (0-8)	–	N/A
ycT category	ycT0−2	3 (15)	15 (31)	0.516
	ycT3ab	7 (35)	18 (37)	
	ycT3cd	4 (20)	6 (12)	
	ycT4ab	6 (30)	10 (20)	
ycN category	ycN0	7 (35)	32 (65)	0.058
	ycN1abc	12 (60)	15 (31)	
	ycN2ab	1 (5)	2 (4)	
Response LLN/tumour deposits	Not applicable	–	36 (73)	0.713
	Complete response	6 (30)	3 (6)	
	Partial response	14 (70)	10 (20)	
Overall response	Good	11 (55)	25 (51)	0.275
	Partial	7 (35)	23 (47)	
	Poor	2 (10)	1 (2)	
*Treatment, primary*				
R status	R0	16 (76)	74 (89)	0.120
	R1	5 (24)	7 (8)	
	Unknown	–	2 (2)	
*LRRC characteristics*				
Nodal recurrence	No	11 (52)	76 (92)	**< 0.001**
	Yes	10 (48)	7 (8)	
	Recurrence	6	7	
	Residue (i.e. after LLND)	4	–	
Predominantly involved compartments	Central + lateral involvement	6 (29)	37 (45)	0.153
	Posterior + lateral involvement	2 (10)	15 (18)	
	Anterior + lateral involvement	–	4 (5)	
	Lateral + involvement other compartment(s)	6 (29)	13 (16)	
	Solitary lateral	7 (33)	14 (17)	
Total number of involved compartments	1	7 (33)	14 (17)	0.113
	2	5 (24)	15 (18)	
	3	2 (10)	28 (34)	
	4	7 (33)	26 (31)	
Hypothesis pathophysiology	Primary tumour	11 (52)	99)	**< 0.001**
	Tumour seeding/spill and/or R1	10	76	
	Tumour deposit	1	–	
	Tumour implant	–	3	
	New lymph node (not LLN)	–	3	
	LLN	10 (48)	1 (1)	

IQR, interquartile range; EMVI, extramural vascular invasion; cT, clinical T stage; cN, clinical N stage; LLN, lateral lymph nodes; SCRT, short-course radiotherapy; mrTRG, MRI tumour regression grade; DWI, diffusion-weighted imaging; ycT, post-neoadjuvant treatment T stage; ycN, post-neoadjuvant N stage; LLND, lateral lymph node dissection; R1, irradical resection margin

*Owing to rounding, not all percentages added up to 100%

LLN+ primary tumours underwent significantly more neoadjuvant CRT (including elective volumes) compared with LLN− primary tumours (95% versus 48%, *p *< 0.001). The proportion of patients receiving induction chemotherapy was similar for both LLN+ and LLN− primary tumours (14% versus 16%, *p *= 0.876). After neoadjuvant therapy, median LLN short-axis size decreased from 10 (IQR 8−12) mm at baseline to 4 (IQR 0–8) mm (Supplementary Fig. 1). In total, 7/21 patients with LLN+ primary tumours underwent LLND, with a median of 8 (IQR 5–12) mm after response evaluation. Radicality of resection did not differ significantly between the LLN+ and LLN− groups (*p *= 0.120).

### Lateral Recurrence: Clinical and Radiological Features

Lateral LRRC disease characteristics are summarised in Table 3. Median interval from primary tumour to lateral LRRC diagnosis was 28 (IQR 17–47) months. Solitary lateral LRRC was observed in 20%. The remaining 80% cases were multi-compartmental, predominantly central and posterior. Vascular involvement/invasion was observed in 45% of cases, multifocal disease in 39% and abscess formation in 12%. On the basis of expert consensus, lateral LRRC attributable to LLN nodal disease was identified in 10/21 (48%) patients with LLN+ primary tumours, including 3 patients who had undergone LLND (Table 2). Among LLN− primary tumours, one case was associated to a LLN identified on primary tumour imaging but classified as non-pathological (SA < 7 mm). Overall, 11/104 (11%) of all lateral LRRC were linked to LLNs visible on primary imaging. The remaining 89% were likely linked to other causes, such as tumour spread or other high-risk features. Of the identified lateral LRRC linked to LLNs, isolated lateral LRRC accounted for 6/11 (55%) and multifocal LRRC accounted for 5/11 (45%).

**Table 3:** Lateral LRRC disease characteristics, treatment and histopathological parameters

		Total (*n *= 104) *N* (%)
Age at diagnosis, recurrence	Mean, years (SD)	64 (10)
Interval primary–LRRC diagnosis	Median, months (IQR)	28 (17–47)
*Baseline MRI, recurrence*		
Maximum diameter tumour	Median, mm (IQR)	36 (22–60)
Multifocal	Yes	40 (39)
Predominantly involved compartments	Central + lateral involvement	43 (41)
	Posterior + lateral involvement	17 (16)
	Anterior + lateral involvement	4 (4)
	Lateral + involvement other compartment(s)	19 (18)
	Solitary lateral	21 (20)
Multifocal, number of lesions	2	18 (17)
	3	17 (16)
	≥ 4	5 (5)
Type of tumour	Solid	84 (81)
	Mucinous	11 (11)
	Fibrotic	7 (7)
	Solid with mucinous component	2 (2)
Abscess formation	Yes	12 (12)
Tumour border	Well defined	26 (25)
	Lobulated or spiculated	35 (34)
	Irregular	43 (41)
Vascular involvement	No	57 (55)
	Vascular involvement	24 (23)
	Vascular invasion	23 (22)
LLN presence, ≥ 7 mm	None	80 (77)
	Single	15 (14)
	Multiple single side	3 (3)
	Both sides	6 (6)
Short-axis LLN	Median, mm (IQR)	10 (8–15)
Lateral compartment involvement	Lateral left	45 (43)
	Lateral right	32 (31)
	Lateral both sides	27 (26)
Therapy, recurrence		
Treatment intent	Curative	80 (77)
	Palliative	24 (23)
Neoadjuvant therapy, curative-intent (*n *= 80)	None	2 (3)
	ICT only	1 (1)
	ICT + chemoradiotherapy	12 (15)
	ICT + chemo-reirradiation	31 (39)
	Chemoradiotherapy	14 (18)
	Chemo-reirradiation	20 (25)
Interval end chemo(re)irradiation and LRRC surgery	Median, weeks (IQR)	13 (11–14)
Surgical procedure, curative (*n *= 66)	LAR	3 (5)
	APR	20 (30)
	Exenterative surgery	23 (35)
	Debulking	20 (30)
Intraoperative electron beam radiotherapy	Yes	57 (86)
Resection margin	R0	46 (70)
	R1	20 (30)
Histology	Adenocarcinoma	58 (88)
	Mucinous carcinoma	2 (3)
	pCR	6 (9)

SD, standard deviation; IQR, interquartile range; LLN, lateral lymph nodes; ICT, induction chemotherapy; LAR, low anterior resection; APR, abdominoperineal resection; R0, clear resection margin; R1, irradical resection margin; pCR, pathological complete response

*Several options possible

**Owing to rounding, not all percentages added up to 100%

### Lateral Recurrence: Treatment Strategies

Most patients undergoing curative-intent therapy at baseline received ICT and chemo(re)irradiation (54%) or neoadjuvant chemo(re)irradiation alone (43%). The majority of surgically treated patients received IOERT (86%). Radical resection was achieved in 70% of cases, and pathological complete response was found in 9%. No operations were aborted intraoperatively on the basis of unresectable tumours.

For patients undergoing palliative care at baseline, treatment modalities consisted of systemic chemotherapy (75%), (chemo)(re)irradiation (20%) or best supportive care (5%).

### Survival Outcomes

The median follow-up was 27 (IQR 19–49) months for patients treated with curative intent and 11 (IQR 7–23) months for patients treated with palliative care. From time since LRRC diagnosis, curative-intent therapy yielded an estimated 1-year OS of 94% and 3-year OS of 66%. Patient receiving palliative care demonstrated an estimated 1-year OS of 57% and 3-year OS of 23% (Supplementary Fig. 2). From time since LRRC surgery, operated patients demonstrated a 1-, 3- and 5-year OS of 89%, 63% and 40%, respectively and 1- and 3-year LRFS of 72% and 38%, respectively. Solitary lateral LRRC demonstrated similar OS (*p *= 0.316) and LRFS (*p *= 0.604) to multi-compartmental lateral LRRC.

## Discussion

The current study characterised features of lateral LRRC and assessed their anatomical relationship to primary tumour features in a large retrospective cohort, following central radiological and expert panel review. The analyses revealed that the primary tumours of lateral LRRC frequently exhibited high-risk radiological features such as EMVI (45%), cT3cd–cT4ab staging (47%) and tumour deposits (23%). Of lateral LRRC, 80% had no LLN+ at the time of the primary tumour. Following expert panel review, less than half of LLN+ primary tumours were subsequently linked to LLN nodal recurrent disease on the basis of radiological assessment. Overall, 11% of lateral LRRC could be attributed to LLN nodal recurrence visible on primary imaging. Conversely, the remaining 89% appeared to be anatomically related to primary tumour spread and other high-risk tumour features.

Current literature reports that lateral LRRC accounts for 18–50% of LRRC.^1–6^ This study demonstrates that isolated solitary lateral LRRC, observed in 20% of cases, is relatively infrequent, with no statistically significant association with LLN+ primary tumours. Instead, lateral LRRC predominantly presents as multi-compartmental disease, with the majority of cases (61%) involving three or four compartments, and a smaller potion (19%) involving two compartments, most frequently centrally and posteriorly. These findings align with Iversen et al., similarly demonstrating frequent involvement of other compartments (central, anterior and posterior involvement in 43%, 35% and 35%, respectively) in lateral LRRC.^4^ While extensive literature regarding the anatomical disease features of lateral LRRC remains limited, these findings suggest that lateral LRRC more often reflects a heterogeneous, complex, infiltrative pelvic disease rather than isolated, lateral unifocal spread.

LLN metastases have been proposed as a pathophysiological driver for lateral LRRC, particularly in locally advanced rectal tumours located below the peritoneal reflection.^12,19^ The international multicentre study by the Lateral Node Consortium reported a 5-year local recurrence rate of 19.5% in patients with LLN+ (SA ≥ 7 mm), despite neoadjuvant (chemo)radiotherapy and TME surgery.^14^ Similarly, the Dutch Snapshot study in 2016, a retrospective cohort of 3057 patients, observed a 4-year lateral LRRC rate of 14.7% in patients with enlarged (≥ 7 mm), compared with 4.4% in those with smaller LLNs (*p *< 0.001).^15^ Both studies identified enlarged LLNs as independent predictors of local recurrence on multivariable analyses. Nonetheless, literature remains limited regarding the extent to which lateral LRRC is likely attributable to LLN involvement versus other primary tumour factors. In our study, the MRI-based prevalence of LLN+ in primary tumours at baseline in a cohort of lateral LRRC was 20%. Comparative data are lacking for the prevalence of LLN+ in primary tumours recurring in other pelvic compartments. However, the observed LLN+ prevalence aligns with previously reported pathological rates of 15–20% in primary rectal cancer.^20–24^ For preoperative identification of LLN metastasis, pelvic MRI is the standard imaging modality with a pooled sensitivity of 0.88.^25^ The radiological MRI-based prevalence of LLN+ varies widely, with retrospective series reporting rates between 9% and 26%.^26–32^ In prospective cohorts, such as the OPRA study, 57 out of 324 (18%) patients had LLN+ at baseline,^33^ which was also seen in the MERCURY study, in which 16 out of 96 (17%) patients had LLN+.^26^ Overall, the prevalence of LLN+ in primary tumours observed in this study was comparable to that reported in pathological and imaging studies. This may suggest that LLN+ may be more a marker of aggressive tumour biology than a primary driver, as the latter would likely show a higher prevalence in this selected population. Nonetheless, direct comparisons between studies that investigated primary rectal cancer and our study with a selected LRRC population are in part limited by differences in clinical context. This study demonstrated that other features such as EMVI (45%), cT3cd–cT4ab (47%), MRF involvement/invasion (46%) and tumour deposits (23%) were more frequently present in primary tumours compared with LLN+. While these high-risk features are well-established as adverse prognostic factors for survival,^18,34–41^ their predictive value specifically for lateral pelvic recurrence has not been widely reported in literature. However, given that the majority of lateral LRRC in this cohort were multi-compartmental, it is plausible that such factors contribute significantly alongside determinants such as tumour cell seeding and irradical resection margins.^42,43^

The current study demonstrated favourable OS among surgically treated patients with lateral LRRC, with a 3- and 5-year OS of 63% and 40%, respectively. Previous retrospective studies have reported estimated 3-year OS for the lateral LRRC populations ranging from 39% to 46%.^4,44^ As these studies predate current practice, the favourable outcomes observed may reflect the cumulative result of improved patient selection, enhanced multidisciplinary care and intensified multimodality therapy.^45^ Currently, the oncological benefit of pelvic sidewall control by prophylactic or selective LLND remains controversial.^16^ While neoadjuvant CRT followed by LLND during TME reduces local recurrence risk, there is no significant impact observed on DFS or OS,^46^ at the cost of long-term sexual and urinary dysfunction.^47,48^ The OPRA study further demonstrated that LLN involvement had no significant impact on LRFS, DFS or distant metastasis-free survival.^33^ Moreover, disease recurrence in LLN was more often accompanied by distant metastases, underscoring that the limited potential benefits of LLND are generally outweighed by the considerable morbidity of the procedure.^33^ In this study, only seven patients underwent LLND, primarily performed in referral institutions, and therefore no robust conclusions can be drawn from out data regarding the efficacy and quality of this procedure. Further research is warranted to refine patient selection and morphological LLN criteria, particularly in the era of neoadjuvant therapy, radiotherapy dose escalation and rectum preservation.

The strengths of this study include the use of protocolised, centrally re-reviewed radiological assessment across a large cohort of lateral LRRC. However, several limitations should be acknowledged. Given the retrospective nature, the study carries inherent selection bias and some clinical data may be incomplete. Nonetheless, all radiological data were complete and re-reviewed, and there is no indication that the missing clinical data would significantly change current results. The heterogeneity in neoadjuvant treatments for the primary tumour across the study population represents a limitation. Nonetheless, it mirrors clinical practice and adheres to the Dutch clinical guidelines applicable during the respective time periods. Additionally, considering the study design, the data are unable to support conclusions regarding the risk of lateral LRRC development in LLN+ primary tumours. Lastly, the small number of LLN+ cases precluded multivariable analyses owing to the risk of model overfitting. Future research efforts should prioritize risk stratification models that integrate tumour biology and anatomical spread, to optimize neoadjuvant treatment pathways and subsequently develop more effective strategies for pelvic sidewall disease.

## Conclusions

Lateral LRRC most often presents as multi-compartmental disease and appears to arise from multifactorial aetiology. Although pathological LLNs in the primary tumour were present in 20% of all lateral LRRC, only 11% of all lateral recurrences could be directly attributed to LLN nodal disease on imaging review. Notably, LLN-related recurrence occurred even after LLND. These findings suggest that LLN metastases constitute just one of several pathways contributing to lateral recurrence development, and that LLN metastases, along with other tumour characteristics such as EMVI, advanced T-stage and tumour deposits, are indicative of aggressive tumour biology. Future studies should develop and validate risk models that incorporate tumour biology and anatomical spread to better guide (neoadjuvant) treatment decisions.

## Supplementary Information

Below is the link to the electronic supplementary material.

Supplementary Fig. 1: Observed changes LLN diameter (short axis, mm) after neoadjuvant therapy for primary rectal cancer. (TIFF 46 kb)

Supplementary file2 (DOCX 67 kb)

Supplementary file3 (DOCX 22 kb)
