# Supplementary figures and images for: Locally Recurrent Rectal Cancer in the Lateral Compartment: Imaging Features and Association with Primary Tumour Characteristics
Source: Ann Surg Oncol. 2026 Jan 22;33(5):3836–48. doi: 10.1245/s10434-025-19068-w (PMC13083428; doi:10.1245/s10434-025-19068-w)

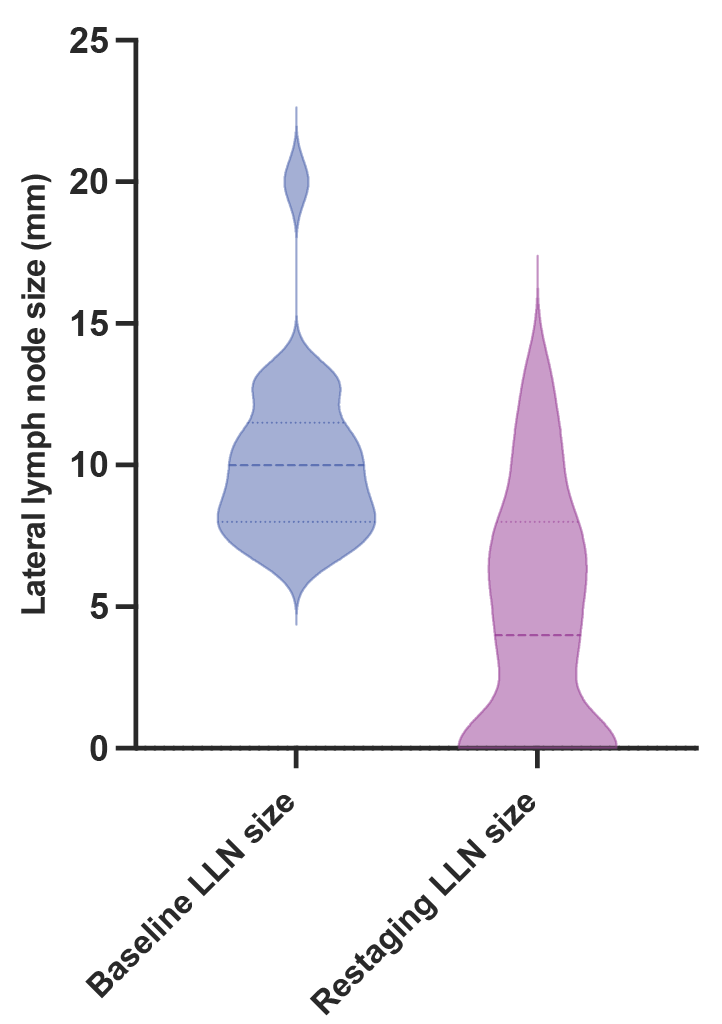

Supplement: Supplementary file 1 — Supplementary Fig. 1: Observed changes LLN diameter (short axis, mm) after neoadjuvant therapy for primary rectal cancer. (TIFF 46 kb) [file 10434_2025_19068_MOESM1_ESM.tiff]
