# Supplementary material for: Locally Recurrent Rectal Cancer in the Lateral Compartment: Imaging Features and Association with Primary Tumour Characteristics
Source: Ann Surg Oncol. 2026 Jan 22;33(5):3836–48. doi: 10.1245/s10434-025-19068-w (PMC13083428; doi:10.1245/s10434-025-19068-w)
Supplement: Supplementary file 2 — Supplementary file2 (DOCX 67 kb) [file 10434_2025_19068_MOESM2_ESM.docx]

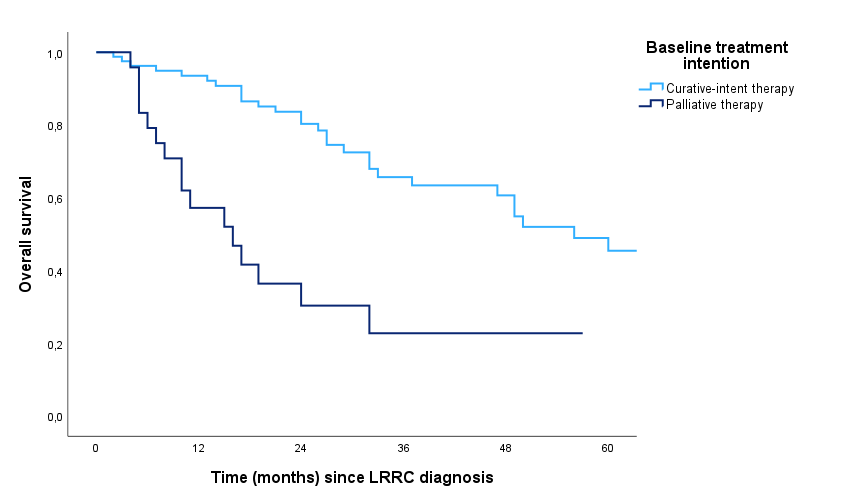


No. at risk (events):
Curative therapy 80 (0) 67 (5) 46 (14) 29 (21) 21 (23) 13 (28)
Palliative therapy 24 (0) 11 (10) 5 (15) 3 (16) 3 (16) 0 (16)

**SUPPLEMENTARY FIGURE 2.** Kaplan-Meier analysis for overall survival in patients with lateral LRRC based on curative-intent therapy versus palliative therapy.
