# Supplementary material for: Locally Recurrent Rectal Cancer in the Lateral Compartment: Imaging Features and Association with Primary Tumour Characteristics
Source: Ann Surg Oncol. 2026 Jan 22;33(5):3836–48. doi: 10.1245/s10434-025-19068-w (PMC13083428; doi:10.1245/s10434-025-19068-w)
Supplement: Supplementary file 3 — Supplementary file3 (DOCX 22 kb) [file 10434_2025_19068_MOESM3_ESM.docx]

**Supplementary material file 1: Standardised reporting protocol**

| **Feature** | **Details** |
| --- | --- |
| Size | Maximum diameter tumour (in mm) |
| Type | Mucinous/Solid tumour/In combination with fistula/abscess |
| Involved compartments | Central/anterior/posterior/lateral/infralevator |
| Region | Anorectal junction (cm) and relation to the sigmoid take-off |
| T stage | cT1: invasion submucosa only  cT2: invasion muscularis propria  cT3: extension beyond the bowel wall into the mesorectal fat.   - cT3a: <1mm extension of infiltration beyond muscularis propria - cT3b: 1-5mm extension beyond muscularis propria - cT3c: 5-15mm extension beyond muscularis propria - cT3d: >15mm extension beyond muscularis propria   cT4: tumour invasion into other organs/structures and/or perforation of the visceral peritoneum   - cT4a: invasion into peritoneum or peritoneal reflection - cT4b: invasion other organs or structures outside the meso-rectum. |
| N stage | cN0: no suspicious lymph nodes  cN1: 1-3 suspicious regional (peri-rectal) lymph nodes   - cN1a: 1 suspicious regional lymph node - cN1b: 2-3 suspicious regional lymph nodes - cN1c: 1-3 suspicious regional lymph nodes and presence of one or more regional tumour deposits   cN2: 4 or more suspicious regional lymph nodes   - cN2a: 4-6 suspicious regional lymph nodes - cN2b: 7 or more suspicious regional lymph nodes - cN2c: 4 or more suspicious regional lymph nodes and presence of one or m ore tumour deposits   Assessment suspicious mesorectal lymph nodes:   - Short-axis 5-8mm in combination with minimum of 2 characteristics:   - Irregular border   - Heterogeneous signal intensity   - Round shape - Short-axis <5mm, in combination with all three aforementioned malignant morphological characteristics - Short-axis ≥9mm |
| Mesorectal fascia (MRF) involvement | Description of circumferential margin (CRM) and invasion or involvement of the MRF of a cT4 tumour. |
| Assessment extramural venous invasion (EMVI) | Negative:   - Grade 0: pattern of tumour extension through the rectal wall is not nodular; no adjacent vessels. - Grade 1: minimal extramural stranding; no adjacent vessels. - Grade 2: stranding in proximity of vessels but no tumour signal in normal calibre lumen. - Grade 3: intermediate signal in lumen of vessels; slight vessel expansion.   Positive:   - Grade 4: irregular vessel contour; definite tumour signal |
| Assessment tumour deposits | - No (residual_ normal lymph node recognizable - Irregular border - In the course of vessels and interrupted vessels - Expansion in vascular structure (vascular trail) - Located separately from the primary tumour |
| Assessment extramesorectal (lateral) lymph nodes | - Short-axis ≥7mm - Location: iliacus internus, iliacus externus or obturator compartment |
| Resectability | Assessment of expected resectability. |

(1) MRI assessment of primary rectal cancer:
*Baseline, at diagnosis*

*Restaging after neoadjuvant therapy*

| **Feature** | **Details** |
| --- | --- |
| Repeated assessment as presented in the baseline template with ycTN stadium and assessment of lateral lymph nodes and tumour deposits (based on size: stable/increase/decrease). | |
| Tumour response | T2W-MRI, according to mrTRG   - mrTRG 1: complete regression (absence of tumour signal and barely visible treatment related scar) - mrTRG 2: good regression (>75% fibrosis, predominant low signal intensity fibrosis with no obvious areas of intermediate signal intensity) - mrTRG 3: moderate regression (50% tumour, 50% fibrosis) - mrTRG 4: slight regression (less than <25% fibrosis, tumour signal intensity dominant) - mrTRG 5: no regression (no fibrosis, same appearances as original tumour)   Response on DWI   - 1: Evident complete response - 2: Complete response very likely - 3: Tumour residue likely, possible complete response - 4: Tumour residue very likely - 5: Tumour residue evident with certainty   Overall response:   - Good response - Partial response - None to poor response |

| **Feature** | **Details** |
| --- | --- |
| Focality | Number of lesions |
| Size | 3 largest lesions: greatest size in mm, measured in any plane |
| Type | Mucinous/Solid tumour/Fibrotic/Other (specify) |
| Border | Well defined / lobulated or spiculated / irregular |
| Venous invasion (mrVI) | Yes/no; if yes: length in mm |
| Lymph nodes: | Yes/no; if yes: central/lateral/inguinal/para-aortal, number of nodes ≥8mm, largest short-axis diameter |
| Abscess formation | Yes/no |
| Previous surgery | LAR, APR, other |
| Primary compartment involved | Central/anterior/posterior/lateral |
| Compartments: central | Involved organs / structures   - Anastomosis: yes / no / not applicable - Levator muscle: left / right / both / no - Puborectalis muscle: left / right / both / no - Sphincter complex: yes / no - Gluteal muscle: left / right / both / no - Perineum: yes / no |
| Compartments: anterior | Involved organs / structures   - Vagina: anterior wall / posterior wall / introitus / portio / no / not applicable - Cervix: yes / no / not applicable - Uterus: yes / no / not applicable - Ovaries: left / right / both / no / not applicable - Prostate: yes / no / not applicable - Seminal vesicles: left / right / both / no / not applicable - Urinary bladder: dome / trigonum / no / not applicable - Peritoneum: yes / no - Small bowel: yes / no - Colon: yes / no - Omentoplasty: yes / no / not applicable |
| Compartments: posterior | Most cranial extension: L5 / S1 / S2 / S3 / S4 / S5 / Coccyx Involved organs/structures   - Presacral fascia: yes / no - Periost/cortex: yes / no - Marrow: yes / no - Neuroforamen S1-S2: yes / no - Neuroforamen S3-S5: yes / no |
| Compartments: lateral | Height: above piriform muscle / at the piriform muscle / below the piriform muscle  Location: mainly inside Lateral Pelvic Fascia (LPF) / at LPF / mainly outside LPF  Involved organs/structures   - Ureter: left / right / both - Sciatic nerve: yes / no - Lumbosacral plexus: yes / no - S1-S2 nerve roots: yes / no - S3-S5 nerve roots: yes / no - Sacrospinous ligament: left / right / both / no - Piriformis muscle: left / right / both / no - Obturator compartment: left / right / both / no |
| Vasculature | Common iliac vein: left / right / both / no  Common iliac artery: left / right / both / no  Internal iliac vein: left / right / both / no  Internal iliac artery: left / right / both / no  External iliac vein: left / right / both / no |

(2) MRI assessment of locally recurrent rectal cancer
*Baseline, at diagnosis:*
